# Phylogenetic reconstruction and the identification of ancient polymorphism in the Bovini tribe (Bovidae, Bovinae)

**DOI:** 10.1186/1471-2164-10-177

**Published:** 2009-04-24

**Authors:** Sean MacEachern, John McEwan, Mike Goddard

**Affiliations:** 1 Primary Industries Research Victoria, Animal Genetics and Genomics, Attwood VIC 3049, Australia; 2 Latrobe University, Department of Genetics, Bundoora VIC 3086, Australia; 3 Animal Genomics, AgResearch, Private Bag 50034, Mosgiel, New Zealand; 4 Melbourne University, School of Agriculture and Food Systems, Melbourne VIC 3000, Australia; 5 Avian Disease and Oncology Laboratory 3606 E Mt Hope Rd, East Lansing, Mi. 48823, USA

## Abstract

**Background:**

The Bovinae subfamily incorporates an array of antelope, buffalo and cattle species. All of the members of this subfamily have diverged recently. Not surprisingly, a number of phylogenetic studies from molecular and morphological data have resulted in ambiguous trees and relationships amongst species, especially for Yak and Bison species. A partial phylogenetic reconstruction of 13 extant members of the Bovini tribe (Bovidae, Bovinae) from 15 complete or partially sequenced autosomal genes is presented.

**Results:**

We identified 3 distinct lineages after the Bovini split from the Boselaphini and Tragelaphini tribes, which has lead to the (1) Buffalo clade (*Bubalus *and *Syncerus *species) and a more recent divergence leading to the (2) Banteng, Gaur and Mithan and (3) Domestic cattle clades. A fourth lineage may also exist that leads to Bison and Yak. However, there was some ambiguity as to whether this was a divergence from the Banteng/Gaur/Mithan or the Domestic cattle clade. From an analysis of approximately 30,000 sites that were amplified in all species 133 sites were identified with ambiguous inheritance, in that all trees implied more than one mutation at the same site. Closer examination of these sites has identified that they are the result of ancient polymorphisms that have subsequently undergone lineage sorting in the Bovini tribe, of which 53 have remained polymorphic since *Bos *and *Bison *species last shared a common ancestor with *Bubalus *between 5–8 million years ago (MYA).

**Conclusion:**

Uncertainty arises in our phylogenetic reconstructions because many species in the Bovini diverged over a short period of time. It appears that a number of sites with ambiguous inheritance have been maintained in subsequent populations by chance (lineage sorting) and that they have contributed to an association between Yak and Domestic cattle and an unreliable phylogenetic reconstruction for the Bison/Yak clade. Interestingly, a number of these aberrant sites are in coding sections of the genome and their identification may have important implications for studying the neutral rate of mutation at nonsynonymous sites. The presence of these sites could help account for the apparent contradiction between levels of polymorphism and effective population size in domesticated cattle.

## Background

The Bovine genome project  has provided researchers with an excellent resource for bovine phylogenetic studies. Genomic resources from *B. taurus *should have some transferability among a number of closely related bovids due to the likely divergence ages of the Bovidae [1]. To date several studies have suggested that primers designed in one species have a relatively high rate of PCR success in other families from the suborder Ruminantia [2,3]. Therefore, sequencing exonic regions should have a high rate of success within the Bovini tribe even from primers designed from the *B. taurus *genome.

### Bovinae subfamily relationships and phylogenetic reconstruction

The Bovidae family consists of some of the most culturally and economically important species on the planet. Of the major representatives of the family Bovidae arguably the most important would be the subfamily Bovinae, which is divided into three main tribes (Table 1); the first two representatives of the Bovinae, the tribes Tragelaphini and Boselaphini, comprise spiral, four-horned and much of the large ox-like antelope [1]. Representatives of these tribes are often hunted for meat and hide, and in the case of Eland have been occasionally used to work in harness. The Bovini tribe comprises all of the major domestic bovine species, including a number of wild species, some of which are endangered or threatened with extinction [1,4-6]. The first divergence within the Bovini occurred between 5–10 million years ago (MYA) with the splitting of the buffalo or the subtribe Bubalina (*Bubalus *and *Syncerus *spp.) from the nonbuffalo or the subtribe Bovina (*Bos *and *Bison *spp.) [1,7-12]. These two subtribes consistently resolve themselves as dichotomous groups, and show no evidence of producing viable hybrid offspring [1,6,9,13,14]. Recently molecular methods have estimated the Bovina Bubalina split at approximately 14 MYA [15]. However, the earliest Bovini fossils found south of the Himalayas in India and Pakistan have been dated at < 9 MYA [16]. Phenotypic and stable carbon and oxygen isotope analysis suggest that these Bovini representatives were behaviourally and ecologically intermediate to modern Bovini and their progenitors the Bosephalini [17]. These early examples of the Bovini appeared to become more adapted to drier more open habitats and were only beginning to develop affinities with grazing open grasslands and obligate drinking patterns [17]. In addition early Bovini fossils do not exist in nearby regions like Afghanistan and Iran suggesting that these early Bovini were restricted to the open forests of India and Pakistan until < 7 MYA when the earliest Bovini fossils begin to appear in Africa, Asia and Europe, respectively. This expansion coincides with a changing climate and expansion of C_4 _dominated grasslands [16-18]. Hence estimates of the Bovinae Bubalina split > 10 MYA are most likely overestimates and a more realistic estimate is < 10 MYA. Within the Bovina subtribe, divergence of the remaining species also occurred recently, with a very sudden radiation in the early Pleistocene ~2 MYA and as a result speciation has not been complete, with many members of the tribe producing viable hybrid offspring or infertile offspring in bulls that may have fertility restored by repeated backcrossing [1,4-6,19]. The dramatic range expansion of domestic cattle has resulted in an increased threat of introgression by domestic cattle DNA into the genomes of many wild populations, either intentionally or accidentally [11,20], all of which may confuse phylogenetic relationships.

**Table 1:** Summary of the representatives from the Subfamily Bovinae

Subfamily	Bovinae			
Tribe	Tragelaphini	Boselaphini	Bovini	
Subtribe	-	-	Bubalina	Bovina
Representatives	Spiral horned and Ox like antelope	Four horned antelope and Nilgai	Buffalo (Bubalus & Syncerus)	Oxen (Bos & Bison)

Another confounding factor when reconstructing phylogenies may arise if a rapidly evolving clade's most recent common ancestor (MRCA) was highly polymorphic, as this may result in the random assortment of genetic variation in different lineages and aberrant modes of inheritance for certain polymorphisms. This phenomenon is known as lineage sorting, and it has been known to cause random associations among species, which may further confuse derived taxonomic relationships [21].

Recently phylogenetic reconstruction using mitochondrial lineages has led to clearer Bovina phylogenies [6,11,15]. However, because mitochondrial DNA is maternally inherited, relationships driven by male mediated introgression may be missed. This finding was confirmed with Y-chromosomal phylogenies identifying close relationships between Wisent and North American bison, while mitochondrial phylogenies identify an association between *Bos taurus *and Wisent [11]. More recently the Kouprey and Banteng were shown to be both valid species despite the introgression of the Kouprey mitochondrial genome in Cambodian Banteng [15]. Thus, some improvements have been made regarding the phylogenetic relationships of the Bovinae subtribe, but there is still concern over the accuracy of many trees due to low confidence values and contrasting results from different data and phylogenetic methods. This is especially evident when considering the position of the Wisent in the bovine phylogeny and the time of divergence for these species.

Despite concerns over the level of variation within nuclear gene sequences at the species level in the Bovini [22,23], by examining a number of nuclear genes (exons and flanking noncoding regions) we should identify enough genetic variation to differentiate between species, populations or breeds. Also by examining variation within genes and exons in closely related species, one can analyse haplotypes for similarity among species over short (variation per amplicon) and large distances (variation between exons and genes). The presence of identical, long haplotypes in different species may be evidence of recent introgression as recombination is likely to break up linked haplotypes after a number of generations. Alternatively, associations generated by the random assortment of polymorphic alleles should not create similar patterns of linked haplotypes over any significant distance. Therefore, lineage sorting and introgression can be differentiated by the analysis of haplotype similarity and sequence variation in genomic DNA between species.

The presence of alleles undergoing lineage sorting in the coding regions of the genome are of interest as their presence is most likely due to polymorphisms in a common ancestor that were sorted in a fashion that is inconsistent with the inferred phylogeny. In some cases these polymorphisms can persist for generations, even past the lifespan of a species. The fact that many of these polymorphisms do not appear to be linked over any large distances suggests they have been independently assorted and that they are ancient in origin and therefore not introduced by recent introgression. If overdominance, selection or demographic factors, like the fixation of slightly deleterious alleles in small populations, have been responsible for the assortment of these ancient polymorphisms they must have an effect on phenotype and should therefore show high dN/dS ratios. Therefore, examining estimates of dN/dS at these sites should help infer whether they are neutral or nearly neutral and if so, whether they are useful for determining the neutral rate of mutation in the Bovini.

In this paper we attempt to reconstruct phylogenies between 14 extant representatives of the Bovinae subfamily. We examined 84 autosomal gene sequences, from 15 different genes, sampled over 4 Bovine chromosomes. Neighbour joining trees and the neutral substitution rate per site at synonymous (dS) and noncoding (dI) regions are presented for all autosomal sequences in an effort to identify relationships and divergence times between species, and test for evidence of introgression or lineage sorting.

## Results

### Sequencing

In total 84 amplicons from 15 genes were sequenced across 14 representatives of the Bovinae subfamily, including members of the Bovini and Tragelaphini tribes, which we used to infer the sequence from the most recent common ancestor (see Methods). From the 84 amplicons 83 amplified in at least one sample. The relative proportions from the amplicons that successfully amplified in each species were particularly high, ranging from 0.76 in Eland to 0.95 in Gaur (additional file 1). Surprisingly some of the wild relatives showed better amplification (Gaur, Yak, Mithan, Banteng and Bison) than different breeds of *B. taurus *(Holstein and Tuli). All sequences were submitted to Genbank and their primers and accession numbers are also available in additional file 1.

### Genetic distances, divergence times and phylogenetic reconstruction

Both dI and dS are generally considered neutral substitutions and are often used for discriminating between phylogenetic lineages. Table 2 shows pairwise comparisons for dI and dS between 15 Bovinae representatives, lower and upper diagonal respectively. The minimum dI is within breeds of Indian river buffalo, followed by breeds of Domestic cow. The largest dI is between species like Eland and the Domestic cow (Table 2, lower diagonal). Similarly, the minimum dS is found between breeds, and the maximum dS is between Eland and other members of the Bovidae, such as Bison, Gaur, Mithan, and Domestic cow, respectively (Table 2, upper diagonal). Interestingly, dS is often greater than dI. Subramanian and Kumar [24] suggest that an elevation in GC content at synonymous sites may be responsible for this phenomenon.

**Table 2:** Pairwise comparisons between members of the Bovini tribe for the number of silent substitutions per site summarised for all genes, with intronic substitutions (dI) below the diagonal and synonymous substitutions (dS) above the diagonal.

	Anc	Ban	Bis	BubB	BubC	Ela	Gau	Her	Ind	Mit	Mur	Hol	Syn	Tul	Yak
Anc	-	**0.023**	**0.025**	**0.033**	**0.032**	**0.032**	**0.026**	**0.021**	**0.032**	**0.024**	**0.034**	**0.02**	**0.028**	**0.023**	**0.024**
Ban	0.016	-	**0.01**	**0.033**	**0.033**	**0.054**	**0.012**	**0.011**	**0.032**	**0.011**	**0.033**	**0.009**	**0.029**	**0.011**	**0.009**
Bis	0.015	0.008	-	**0.034**	**0.035**	**0.06**	**0.012**	**0.011**	**0.034**	**0.013**	**0.033**	**0.01**	**0.031**	**0.01**	**0.009**
BubB	0.018	0.023	0.022	-	**0.005**	**0.057**	**0.034**	**0.034**	**0.006**	**0.033**	**0.004**	**0.032**	**0.02**	**0.034**	**0.033**
BubC	0.018	0.022	0.022	0.004	-	**0.053**	**0.035**	**0.034**	**0.003**	**0.034**	**0.003**	**0.033**	**0.02**	**0.035**	**0.035**
Ela	0.024	0.038	0.038	0.037	0.037	-	**0.059**	**0.058**	**0.058**	**0.059**	**0.055**	**0.053**	**0.057**	**0.059**	**0.059**
Gau	0.016	0.008	0.008	0.022	0.022	0.038	-	**0.012**	**0.035**	**0.006**	**0.034**	**0.011**	**0.031**	**0.013**	**0.009**
Her	0.016	0.011	0.01	0.024	0.023	0.04	0.012	-	**0.034**	**0.01**	**0.035**	**0.002**	**0.029**	**0.003**	**0.01**
Ind	0.019	0.023	0.024	0.005	0.003	0.038	0.025	0.028	-	**0.034**	**0.002**	**0.032**	**0.02**	**0.034**	**0.034**
Mit	0.016	0.008	0.008	0.022	0.022	0.04	0.006	0.009	0.023	-	**0.034**	**0.008**	**0.03**	**0.01**	**0.009**
Mur	0.019	0.024	0.024	0.004	0.004	0.038	0.025	0.025	0.003	0.024	-	**0.032**	**0.019**	**0.035**	**0.034**
Hol	0.015	0.009	0.008	0.023	0.022	0.039	0.011	0.003	0.025	0.009	0.024	-	**0.026**	**0.003**	**0.008**
Syn	0.016	0.023	0.022	0.017	0.016	0.037	0.023	0.024	0.018	0.023	0.018	0.024	-	**0.029**	**0.028**
Tul	0.016	0.01	0.009	0.023	0.023	0.039	0.011	0.004	0.025	0.009	0.025	0.003	0.024	-	**0.009**
Yak	0.016	0.008	0.006	0.022	0.022	0.038	0.008	0.008	0.024	0.008	0.023	0.007	0.022	0.007	-

Species: (Anc: Ancient, Ban: Banteng, Bis: Bison, BubB & BubC: Asian water buffalo, Ela: Eland, Gau: Gaur, Her: Hereford, Ind & Mur: Indian water buffalo, Mit: Mithan, Hol: Holstein, Syn: African buffalo, Tul: Tuli, and Yak)

Table 3 presents the divergence times estimated from the pairwise comparisons between all major lineages and Hereford (*B. taurus*) using Nei's D (equation 5, Methods). Nei has shown that genetic distance (D) is directly related to divergence time. However, the method may be susceptible to inaccurate estimates given the incorrect mutation rate. As a result we also estimated divergence times using a relaxed molecular clock using calibration points based on best estimates of divergence times in the fossil record for Bison and Yak (2.0 and 1.7 MYA, respectively). Members of the Bovini subtribe appear to have diverged from *B. taurus *approximately 2–3 MYA. The Bubalina subtribe has diverged from *B. taurus *some 5–9 MYA, while Eland appears to have diverged from the Domestic cow some 8–14 MYA. The breeds of *B. taurus *show some interesting results with African cattle (Tuli) seeming to have diverged ~100,000–200,000 years ago, which may be possible if African and European cattle have in fact been separately domesticated, or if Tuli contains genes originating in *B. indicus*. However, as more polymorphism has been detected within Holstein than between Hereford and Holstein, there has been some difficulty estimating divergence times between these two closely related breeds, which were most likely separated only a few hundred years ago.

**Table 3:** Estimate of divergence times in millions of years (MY) for pairwise comparisons (dI) between Hereford and members of the Bovinae subtribe using estimates from Nei (T_N_) and a relaxed molecular clock method (T_C_) using Bison and Yak divergence dates from fossil records as calibration points (2.0 and 1.7 MYA, respectively)

	Hol	Tul	Ban	Bis	Gau	Mit	Yak	Anc	BubB	BubC	Ind	Mur	Syn	Ela
T_N_	-0.13	0.1	1.4	1.2	1.6	1.0	0.9	2.4	4.2	3.9	5.0	4.2	4.1	7.4
T_C_	-0.25	0.15	2.6	2.1	3.0	1.9	1.6	4.5	7.7	7.0	9.1	7.9	7.7	13.7

Species: (Her: Hereford, Tul: Tuli, Ban: Banteng, Bis: Bison, Gau: Gaur, Mit: Mithan, Yak, Anc: Ancient, BubB & BubC: Asian water buffalo, Ind & Mur: Indian water buffalo, Syn: African buffalo and Ela: Eland)

No significant differences were detected between overall base frequencies (A, T, C and G) for any member of the Bovini tribe, and substitution rates between Ancestral and Eland sequences with all members of the Bovini appear to be largely the same. A small bias was detected for transition (ti) to transversion (tv) substitutions, with a ratio of ti/tv = 2.1 (results not shown). Therefore, Kimura's two parameter model should be appropriate for phylogenetic reconstruction. However, the relatively small genetic distances detected (dS and dI < 0.05) suggest that the p-distances calculated in table 2 should also be accurate despite the transition bias [25].

**Figure 1:**
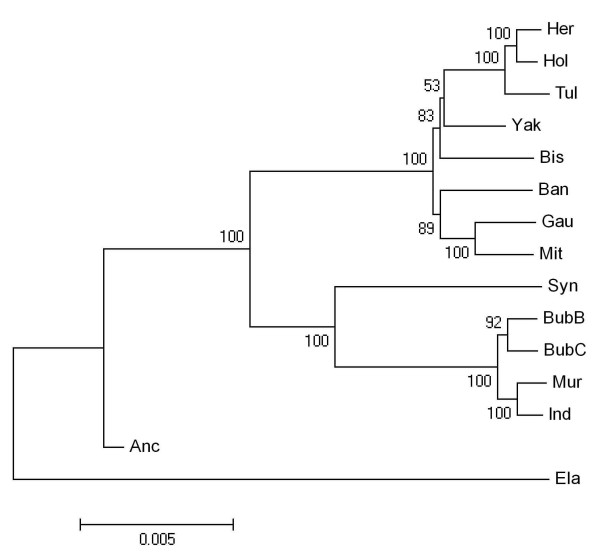
**Neighbour joining analysis for members of the Bovini tribe (Anc: Ancient, Ban: Banteng, Bis: Bison, BubB & BubC: Asian buffalo, water and swamp type, Ela: Eland, Gau: Gaur, Her: Hereford, Ind & Mur: Indian water buffalo, Mit: Mithan, Hol: Holstein, Syn: African buffalo, Tul: Tuli, and Yak) using Kimura two parameter model with bootstrap values (5000 replicates) overlying branchpoints from an alignment of ~30000 bp with 1786 segregating sites**.

Figure 1 shows a monophyletic neighbour joining tree, deduced using Kimura's two parameter model from an alignment of ~30,000 bases and 1,800 variable sites (additional file 2). The placement of the inferred ancestral sequence is not surprising and may indicate its accuracy. Strong support was detected for a bifurcation between representatives of the Bovina and Bubalina subtribes. Within the Bubalina subtribe there is strong support for separation of the *Syncerus *and *Bubalus *genera. Within the water buffalo strong stratification is also apparent. Surprisingly, one of the river buffalo (BubB) has a closer relationship to swamp buffalo (BubC) than to other members of the same species, the separation between buffalo may be associated with geographic patterns as two of our buffalo samples (Ind and Mur) originated from India, while the swamp buffalo are generally restricted to East Asia and are known to hybridise with river buffalo. Within the Bovina subtribe a distinct separation between Bison, Yak and Domestic cattle with the cattle of Indochina (Gaur/Mithan/Banteng) was found. However, the nodes leading to a Bison/Yak/Domestic cattle clade show less support with the Yak/Domestic cattle clade showing less than 60% support by bootstrap resampling. The lack of certainty in the tree is not surprising given the short branch lengths leading to the divergences among Domestic cattle-Yak-Bison-Banteng-Mithan/Gaur. Previous work has placed the lineage leading to Bison and Yak as a separate group, which diverged from the Banteng/Gaur/Mithan clade [6,11], and more recently as a divergence from the *B. taurus *branch [15].

### Aberrant sites

To detect if recent gene flow or ancient polymorphisms were responsible for differences between our and other recently published phylogenies, we analysed all of the segregating sites from figure 1 for the presence of anomalies. We reconstructed a number of phylogenetic trees to identify the tree that minimises the number of aberrant sites, which we defined as any site that must have arisen due to a double mutation or lineage sorting of an ancient polymorphism (see Methods). We identified 133 aberrant sites, of which 111 were not tree specific and were aberrant in all trees leaving 22 informative sites. From the 111 non-tree specific aberrant sites 53 ancient sites are segregating within both the Bovina and Bubalina lineages. A legend to all aberrant sites, their position and the relative age (i.e. approximately how long they have been segregating in the clade) is provided in additional file 3.

To examine these aberrant sites in more detail we have analysed a number of phylogenetic trees that have been suggested due to the poor bootstrapping confidence in figure 1, and trees presented in recent work by Verkaar et al and Hassanin and Ropiquet [6,11]. The phylogenetic tree that generates the smallest number of anomalies (125) places Yak off the lineage leading to Domestic cattle and Bison off the lineage leading to the Indochinese cattle ((B. taurus, Yak), (Bison, Indochinese cattle)); however, similar numbers (126) are also seen for the tree that places Bison and Yak as a lineage of the Indochinese clade ((B. taurus), ((Yak, Bison) Indochinese cattle)), which is only worse by one aberrant site. Therefore, some evidence exists for the trees recently proposed by Hassanin and Ropiquet [6] and Verkaar et al. [11]. However, it appears that a number of aberrant sites are generating an association between Yak and Domestic cattle, which has confounded the phylogenetic reconstruction. Removing all aberrant sites from the analysis leaves fewer substitutions to accurately estimate genetic distances between members of the Bovina subtribe and the tree is less certain (data not shown).

### Introgression and ancient polymorphism

To identify the source of the aberrant substitutions within the Bovini tribe, we compared all variable sites in the alignment to find sites shared with *B. taurus *at normal and aberrant positions (Table 4). We examined these positions within Gaur, Mithan, Banteng, Bison and Yak for similarities to Domestic cattle over extended haplotypes resolved from aberrant and all variable sites. We found that Yak shares the largest number of alleles in common with Domestic cattle for all variable and aberrant sites (Table 4). Perhaps the most interesting finding is the high number of identical haplotypes Yak and Domestic cattle share over haplotypes resolved from the aberrant sites per amplicon (Table 4, Table 5). When compared to haplotypes resolved from the all variable sites, Yak and Domestic cattle share the largest number in common. However, there are relatively few haplotypes that are similar for any of the species (Gaur, Mithan, Banteng, Bison and Yak) and Domestic cattle, and this is probably a function of the species specific mutations that have accumulated per amplicon since divergence from the MRCA.

**Table 4:** Summary of the number of substitutions in the total alignment that are the same as *Bos taurus *compared between species at all variable sites (n = 1786), and at all aberrant sites (n = 133).

	Similar Subs (All sites)	Similar Subs (Aberrant sites)	Haplotypes (All sites)	Haplotypes (Aberrant sites)
Gaur	888	49	3	8
Mithan	891	63	2	12
Banteng	886	69	0	11
Bison	882	74	1	12
Yak	917	79	4	20

And the number of substitutions similar to *B. taurus *over haplotypes generated from all variable and from aberrant sites for each amplicon.

**Table 5:** Summary of taurine haplotypes at aberrant sites for each amplicon determined from Hereford sequence and the species sharing the same haplotype (*).

Gene_Amplicon (Taurine Haplotype)	Chromosome	Gaur	Mithan	Banteng	Bison	Yak
NRIP1_e1.2 (G)	bta01	*	*	*		*
NRIP1_e1.3 (T)	bta01	*	*		*	*
PIT1_e1 (T)	bta01		*	*	*	*
PIT1_e3 (T)	bta01			*	*	*
PIT1_e5 (C)	bta01	*	*	*	*	
PIT1_e6 (ACAGTGAACTCCTAG)	bta01					*
ITGBP5_e3 (GG)	bta01	*				
ITGBP5_e5 (ATAGAC)	bta01					*
ITGBP5_e6 (CGTG)	bta01		*		*	
ITGBP5_e10 (CG)	bta01				*	*
5HT1F_e1 (T)	bta01	*	*			
IGFBP2_e1 (AT)	bta02		*			
IGFBP5_e2 (CGG)	bta02		*			
IGFBP5_e3 (ACGGCC)	bta02		*			
HFABP_e2 (GTTC)	bta02		*			
HFABP_e3 (TGTA)	bta02					*
LACS3_e1 (T)	bta02	*				
LACS3_e3 (T)	bta02					*
LACS3_e4 (C)	bta02			*	*	
LACS3_e6 (CA)	bta02				*	
5HT6_e1.2 (TGTGCA)	bta02				*	
GMEB1_e1 (AC)	bta02					*
GMEB1_e3 (CCGG)	bta02		*			*
GMEB1_e4 (A)	bta02					*
GMEB1_e5 (AC)	bta02				*	
GMEB1_e6 (G)	bta02	*	*			
GMEB1_e7 (TA)	bta02	*			*	*
GMEB1_e8 (CT)	bta02	*	*	*		
EGF_e1 (TTA)	bta06			*		
ERa_e3 (A)	bta09			*	*	
ERa_e4 (C)	bta09					*
ERa_e5 (AC)	bta09					*
ERa_e6 (T)	bta09	*		*		
ERa_e7 (TGA)	bta09					*
PABPC1_e2 (AC)	bta14			*		
PABPC1_e3 (TTTGCG)	bta14					*
PABPC1_e5 (GG)	bta14			*		
PABPC1_e9 (CCCACGCACTAGACAGCG)	bta14					*
MFGE8_e2 (CCCTT)	bta21					*
MFGE8_e3 (ATC)	bta21			*		
MFGE8_e5 (TCCA)	bta21			*		*

*Number of exons represented as e(n) where e = exon and n = number, therefore, PIT1_e1 is exon 1 of PIT1. Exons that extend over the largest sequencing read, which is typically 800 bp are represented as e(n.j) where j equals order of read from 5-prime to 3-prime. Therefore NRIP_e1.1 and NRIP_e1.2 represent two subsequent reads of the l

The number of aberrant alleles making each haplotype are given in brackets as the (taurine haplotype). Table displays haplotype blocks shared with Hereford and 5 members of the Bovinae for exons within the each gene and for genes on the same chromosome.

To determine if the high proportion of haplotypes, resolved from aberrant sites, shared between Yak and Domestic cattle may be evidence of recent introgression, we looked for extended haplotypes that are shared between species. Thus, the extent of linkage between neighbouring amplicons and genes on the same chromosome was examined. Table 5 shows the *B. taurus *haplotype per amplicon from all aberrant sites and the species that share the same haplotype. A number of haplotypes are shared with all species. Many of the haplotypes that are based on one aberrant site (PIT1_e1) may be similar by chance and therefore may not be very informative. However, by extending haplotypes to adjacent exons and genes more power to detect recent hybridisation should be available. But as haplotypes are extended to closely linked amplicons or neighbouring genes on the same chromosome this similarity rapidly disappears (Table 5).

**Figure 2:**
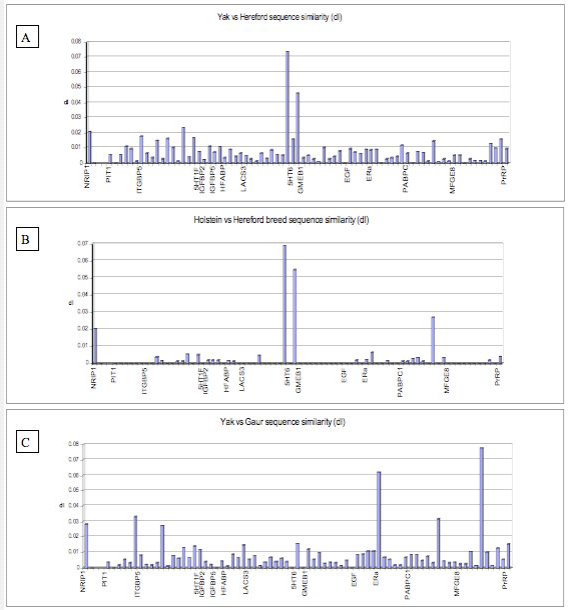
**Plots of sequence similarity for noncoding substitutions per site (dI) for pairwise comparisons between Hereford vs Yak (A), Hereford vs Holstein (B) and Yak vs Gaur (C) for all amplicons all plots are in chromosomal order**.

In a further attempt to identify recent introgression, sequence diversity estimates were examined between neighbouring amplicons and genes. Figure 2 shows plots of sequence divergence at noncoding sites (dI) for Hereford vs Yak (A), Hereford vs Holstein (B) and Yak vs Gaur (C). A similar pattern is seen to that described in table 5. A number of amplicons show similar amounts of sequence divergence between Hereford vs Yak as is seen between Hereford vs Holstein (NRIP1 and PIT1). However, genetic diversity at closely linked amplicons remains low between breeds of *B. taurus *for closely spaced genes on chromosome 1 (PIT1, ITGB5) and chromosome 2 (GMEB1 and EGF), which suggests that these animals have recently shared a common ancestor. In contrast, comparisons for Domestic cattle vs Yak, and Yak vs Gaur (Figure 2A and 2C, respectively) rarely show low sequence diversity over similar linked regions, and thus, genetic associations are most likely not the result of recent introgression.

**Figure 3:**
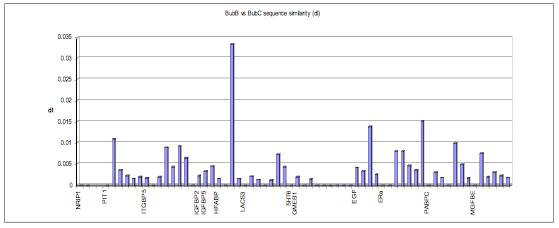
**Plot of sequence similarity (dI) for genes in chromosomal order between BubB (River buffalo) and BubC (Swamp buffalo), showing high degrees of similarity over large regions of chromosomes 1 and 2**.

An examination of the river and swamp buffalo samples from Southeast Asia (BubB and BubC) has identified large segments of chromosome 1 and 2 that are identical. Thus BubB has a closer relationship to swamp buffalo (BubC) than to other members of river buffalo that originate from India, which may suggest some level of introgression in these two samples as large portions of chromosomes 1 and 2 in BubB appear to be of swamp buffalo origin (Figure 3).

### Classifying aberrant sites: age and role of selection

A number of aberrant sites were identified that were inconsistent with any of the phylogenetic trees suggested from either molecular or morphological characteristics. In total 33 aberrant sites occurred in coding regions, of which 6 are Ka and 27 are Ks from 11,563 synonymous and 3,482 nonsynonymous sites, which resulted in a dN/dS ratio of 0.065. From the 33 aberrant sites 10 appear to be polymorphic since the common ancestor with buffalo, while the remaining 23 would have been polymorphic in the common ancestor of the *Bos *and *Bison *genera. Of the 10 ancient polymorphisms 3 are Ka and 7 are Ks. Therefore these 10 ancient polymorphisms were maintained for millions of years. They must have been polymorphic in the MRCA to the Bovina and Bubalina subtribes some 5–8 MYA and appear to have persisted to at least the divergence of the Bovina subtribe around 2 MYA. The small values estimated for the dN/dS ratios calculated suggest that these polymorphisms may have been neutral.

## Discussion

### Sequencing

A high proportion of successful amplification was achieved for all species within the Bovinae subfamily from primers designed using the *Bos taurus *genome despite divergence times of 8–14 MY (*Taurotragus oryx*). The high rate of amplification between species should facilitate further population genetic studies in the Bovini and possibly other ruminant subfamilies.

### Bovine phylogeny reconstruction, mutation and substitution

Within the Bovini, difficulties resolving interspecific relationships arose when comparing pairwise differences between species for dI and dS with those deduced from the neighbour joining algorithm. In general the phylogenetic reconstruction created using Kimura's two parameter method and the neighbour joining algorithm agreed with the p-distances calculated for dI and dS in table 2, with strong separation between Bubalina and Bovina subtribes and strong support for separation of *Bubalus *and *Syncerus *genera, confirming previous studies from molecular data [6,11,26]. Within the *Bubalus *there appears to be a separation between swamp and river buffalo and a possible separation within river buffalo that may be due to due to geographic patterns and/or introgression of swamp and river buffalo. The phylogenetic tree we identified does not agree with some of the individual genetic distances within the Bovina. For instance, the genetic distance between Yak and Bison for dI and dS both imply that the species are most likely a monophyletic group that could either be grouped with the *B. taurus *lineage or the lineage leading to the cattle from Indochina (Gaur/Mithan/Banteng) but the tree places them as separate divergences from the lineage leading to *B. taurus*. This disparity may be the result of more complicated methods of deducing genetic distance, like the Kimura two parameter model, inflating variances over simpler methods, such as the p-distances, thereby increasing the chance of topographical errors [25]. Low bootstrapping levels may be the result of low genetic differentiation between these three lineages and the presence of aberrant polymorphisms, confusing phylogenetic reconstruction.

Examining a number of manually inferred trees that attempt to minimise the number of aberrant polymorphisms identified a tree that has Bison as a divergence from the Gaur/Mithan/Banteng lineage and Yak as a divergence of the *B. taurus *clade. But this relationship does not seem logical considering the close relationship identified between Bison and Yak for dS and dI distances. Therefore, the large number of aberrant sites that Yak shares with Hereford may confuse the phylogenetic reconstruction, as the neighbour joining algorithm deals with these aberrant polymorphisms in the same manner as all other polymorphisms.

Removing aberrant polymorphisms from the analysis identified a phylogenetic relationship in which all members of the Bovina appear to have diverged roughly at the same time in a star like phylogeny. However, bootstrap values were even lower than those deduced from the entire dataset, highlighting the difficulty of reconstructing phylogenies from a group of species that have undergone rapid genetic isolation. Of course, low bootstrap values are expected for a star-like phylogeny.

All trees show a close association between members of the Bovina subtribe, with all major lineages diverging very rapidly some 1–3 million years ago, which appears to be associated with climatic oscillations during the late Pleistocene [27,28]. All trees show that Bison and Yak are single or multiple divergences from the clade leading to *B. taurus*, which disagrees with previous work by Verkaar et al [11] and Hassanin and Ropiquet [6] that place Bison and Yak as a single divergence from the Indochinese cattle. More recently Hassanin and Ropiquet [15] show Bison and Yak as a single divergence from the lineage leading to *B. taurus*. However, these studies were typically based only on one or two thousand nucleotide characters or from a small number of genetic markers and therefore, each tree typically had low bootstrapping values or resulted in variations to this tree when differing methods or maternally, paternally or bi-parentally inherited data are used, suggesting that some of these relationships are still contentious. The phylogenetic reconstruction presented above is based on over 29,000 bi-parentally inherited nucleotide characters and should have more power to resolve some of the more confusing intergenic relationships within the Bovini.

The association identified between Bison, Yak and *B. taurus *may also explain the similarity seen between Wisent and *B. taurus*. Although, no Wisent sample was represented in our study, Verkaar et al [11] and Hassanin and Ropiquet [6] have recently hypothesised that as a result of repeated male mitigated introgression of American bison populations into Eurasian cattle like populations lead to a new species (Wisent), which comprises the phenotype, autosomal genes and Y-chromosome of American bison and the mitochondrial genome from the maternal ancestor (*B. taurus *like species) [11]. Thus, the constant hybridisation by the progenitors of Bison and *B. taurus *may have had a large impact on the genomes of both species.

Surprisingly, one of the *Bubalus bubalis *(river buffalo) samples was more closely related to the *Bubalis carabenensis *(swamp buffalo) than to other river buffalo. This relationship was puzzling, as previous studies have found that the two subspecies are genetically distinct, have different chromosome numbers and their divergence time has been estimated tens of thousands to over one million years [29,30]. However, despite the obvious phenotypic and genetic differences, the two subspecies are known to hybridise, especially in South East Asia where a hybrid zone for buffalo may exist [30,31]. Also in some instances animals can show the phenotype of the swamp buffalo but have the chromosomal and genetic make up of the river buffalo, as is the case for animals in Sri Lanka [32]. Therefore, the phylogenetic reconstruction may have been confused due to an analysis of a hybrid river buffalo (BubB) that has large regions of chromosome 1 and 2 that apparently originated from swamp buffalo. Alternatively, the phenotypic and karyoptypic plasticity shown within water buffalo may indicate that genetic differences between river and swamp buffalo could all be within species polymorphism.

### Aberrant sites, introgression and ancient polymorphism

One of the most astonishing features of the phylogenetic reconstruction was the identification of a large number of aberrant sites that did not fit any known phylogenetic reconstructions. A number of explanations may account for these phylogenetic aberrations, such as double mutations, introgressive hybridisation and lineage sorting. The probability of aberrant sites as a result of double mutations can be calculated as follows:

(1)

which is the probability of no mutation in one lineage with mutation rate μ over t generations. Therefore, the probability of mutation in one lineage is

(2)

Thus the probability that the same site mutates in two different lineages is

(3)

And the expected number of double mutations across n sites is

(4)

The probability that both mutations result in the same base is approximately 1/3. Hence the expected number of double mutations in two lineages is

(5)

Therefore, even if t is set to 10 million years as a conservative estimate for the divergence of *B. taurus *and *B. bubalis *and n = 30,000 then for equation 5 one would expect around 2 bases to have undergone a double mutation resulting in the same derived allele from the ~30,000 bases sequenced in each lineage since the divergence of *B. taurus *and *B. bubalus *from a common ancestor some 10 MYA. The time of divergence is somewhat less within the Bovini but there are more than 2 lineages. However, the likelihood of a mutation affecting the exact same base ~130 times in our dataset is highly improbable, so in general, most abnormalities must be evidence of lineage sorting or introgressive hybridisation and not double mutations.

Also a high proportion (40%) of aberrant sites were identified as sites that were deemed to be polymorphic in Bubalina and Bovina subtribes. Members of these two subtribes separated 5–8 MYA and no hybridisation has ever been documented between any members of these two subtribes [1]. Therefore, these aberrant sites cannot have arisen due to recent hybridisation events. If 40% of the aberrant sites are due to polymorphisms that existed in the common ancestor at Bubalina and Bovina, it seems likely that other polymorphisms in the common ancestor of Bovina will also have contributed to the other 60% of aberrant sites. However, recent introgression might have also contributed, as the incomplete speciation of the tribe has resulted in many viable hybrid offspring from various crossings [1].

The presence of identical haplotypes between Bovina representatives and Domestic cattle may also be a result of ancient geneflow between animals before reproductive isolation was achieved, but in essence this hypothesis would virtually be identical to the presence of ancestral polymorphisms. Another possibility for this finding may also be explained by hybridisation between another closely related species. A previous study from the labs of University Station in Texas also found an anomalous association between Yak and Taurine cattle, due to an animal descending from a *B. indicus *cow [5,9,11]. The fact that a *Bos indicus *sample was not included in the data set, meant tests could not determine if some of the aberrant sites in our sample were due to gene flow between *B. grunniens *and *B. indicus *and this will need to be investigated further.

The high degree of sequence similarity detected between Yak and Domestic cattle at all variable sites in the alignment, aberrant sites and over extended haplotypes suggests that hybridisation may have also occurred between these two species (Table 4). Yak-taurine hybrids are reported in China and Mongolia where their altitudinal ranges intersect [1]. However, no evidence of linked haplotypes at aberrant sites between neighbouring genes or on very small scales between exons within the same gene was detected (Table 5). Genes were selected from tightly linked regions of the *B. taurus *genome and the majority of amplicons within genes were only separated by 1–20 kilobases. No information on the genetic distance between genes has been collected but, the small physical distances between amplicons on the same gene suggest the recombination rate between many of these amplicons would be far below 1% per generation. The fact that all animals rarely share haplotypes with *B. taurus *between neighbouring amplicons and genes suggests that there is no evidence of linked haplotypes. This finding is also reflected in plots of sequence diversity between Yak and Domestic cattle with low sequence diversity detected for some amplicons but consistently larger estimates found for neighbouring amplicons from the same gene (Figure 2A). Thus recent introgression has not played a large role in the presence of these aberrant sites within the Bovina and they may be due to homologous polymorphisms that existed in the MRCA that have undergone lineage sorting in subsequent generations. However, the lack of information on the genetic ancestry for all of our animals may require some caution when interpreting some of our results.

Ancestral polymorphisms that have undergone lineage sorting in the Bovini would have required an ancestral species with an extremely large population size that was very polymorphic. Palaeontological evidence generally agrees with this assumption, with the earliest fossil for the Bovini tribe found in Asia ~8 MYA, with evidence of populations existing solely in Pakistan and India, south of the Himalayas. It was not until about 6–7 MYA that the Himalayas began increasing in size and areas of desert were becoming seasonally humid facilitating wide dispersal of Bovini ancestors [6]. Thus the 52 aberrant sites identified in the Bubalina and Bovina subtribes most likely originate in this once large restricted and panmictic population. The fact that each allele is found in the Bubalina and Bovina suggest that the polymorphisms must have remained segregating in both groups for what would appear to be ~8 million years. The remaining aberrant sites appear to have arisen in the independent ancestors of the Bovina, which suggests that the respective population sizes for these populations must have also been very large. These sites may have persisted for at least 2 million years, which is the approximate divergence time for the major Bovina lineages from molecular and palaeontological data [6,11,27,28,33,34]. The finding that some polymorphisms in the Bovini have been segregating for so long is completely novel. These ancestral polymorphisms have existed for very long periods and been maintained as neutral or overdominant polymorphisms. The low dN/dS among these polymorphisms suggest that they are neutral because it is unlikely the synonymous polymorphisms show overdominance.

Knowledge of polymorphisms segregating in humans and other primates has been known for some time with examples of polymorphisms in ABO, MN and Lewis blood groups [35], although it was not known whether these polymorphisms were the result of the same genetic variations. Recently, work on the MHC has identified a number of polymorphisms that are trans-specific and have existed in humans and other primates for millions of years [36,37]. However, the majority of these genes are involved with disease resistance and the maintenance of polymorphism has been hypothesised to exist due to balancing selection. To our knowledge this is one of the only examples where polymorphisms at the same alleles have been maintained for such long periods outside of the MHC or involving genes that are responsible for disease resistance.

The difficulties in phylogenetic reconstruction caused by lineage sorting in closely related species have previously been reported for neutral genetic markers and mitochondrial control regions [11,22,38]. Because the majority of phylogenetic studies typically only involve a few genetic markers or concentrate on specific regions of the mitochondrial genome the exact rate of alleles undergoing lineage sorting is unknown, especially for nuclear genes, but the maintenance of polymorphic sites beyond the lifespan of a species is expected to be rare [39]. Therefore, the presence of alleles undergoing lineage sorting in coding regions of the bovine genome should be extremely scarce. However, a relatively large number of these aberrant sites were detected within genomic coding regions of the Bovini tribe. These substitutions most likely would have been neutral or nearly neutral in the ancestral population from which they were derived, and thus the probability of their loss and fixation in future lineages is governed by the neutral theory of molecular evolution [40]. By sequencing a large number of close relatives researchers should be able to identify a high number of these aberrant sites and infer the dN/dS rate among neutral mutations.

## Conclusion

In conclusion, a partial phylogenetic reconstruction of the Bovinae subfamily has identified three distinct lineages that have arisen after the Bovini split from the Bosephalaphini and the Tragelaphini tribes. Within the Bovini tribe two subtribes the Bubalina and Bovina were resolved, which we estimate diverged approximately 5–8 MYA. About 2–3 MYA the Bovina subtribe was still a single widespread species. It then appears that the Bovina split into 3 major groups: Domestic cattle, Bison/Yak and the Indochinese cattle or Gaur/Mithan and Banteng and this occurred very rapidly. Polymorphisms in the ancestral species continued to segregate for long periods of time, which has lead to some alleles undergoing lineage sorting. The lineage sorting we have detected appears to have confused the algorithms and tree drawing programs used for this analysis, and the apparent star like phylogeny will most likely continue to confuse phylogenetic reconstructions. The majority of these sites appear to be neutral and must have arose in a polymorphic ancestor and continued to segregate within species for millions of years. The age and maintenance of these ancestral polymorphisms in coding regions may be useful for studying the neutral rate of evolution and could also help explain the discrepancies between effective population size and levels of polymorphism in domesticated cattle.

## Methods

### Gene candidates

Candidate genes were selected for sequencing using *a priori *knowledge of the gene's position in relation to suspected milk QTL on chromosomes 1, 2, 4 and 9. In addition MacEachern et al. have compared a large number of genes and EST for evidence of higher than average rates of molecular evolution between *Bos taurus*, *Homo sapiens *and *Bos indicus *[41,42]. Genes showing higher than average rates of molecular evolution between these species may be of evolutionary importance and are good candidates to study molecular evolution. This potential list of genes was further reduced by selecting genes that might have a role in milk production from examination of the most recent annotation of the human genome. Table 6 summarises the genes, descriptions and their chromosomal location from the latest cattle genomic resources.

**Table 6:** Summary of the genes sequenced for the phylogenetic reconstruction

Common Name	Chromosome	Description
NRIP1	bta01	Nuclear factor RIP140 (Nuclear receptor interacting protein 1)
PIT1	bta01	Pituitary-specific positive transcription factor 1 (Pit-1) (Growth hormone factor 1) (GHF-1)
ITGBP5	bta01	Integrin beta-5 precursor.
5HT1F	bta01	5-hydroxytryptamine 1F receptor (5-HT-1F) (Serotonin receptor 1F)
IGFBP2	bta02	Insulin-like growth factor binding protein 2 precursor (IGFBP-2)
IGFBP5	bta02	Insulin-like growth factor binding protein 5 precursor (IGFBP-5)
HFABP	bta02	Fatty acid-binding protein, heart (H-FABP) (Heart-type fatty acid – binding protein) (Muscle fatty acid-binding protein) (M-FABP) (Mammary-derived growth inhibitor) (MDGI)
LACS3	bta02	Long-chain-fatty-acid – CoA ligase 3 (EC 6.2.1.3) (Long-chain acyl-CoA synthetase 3) (LACS 3)
5HT6	bta02	5-hydroxytryptamine 6 receptor (5-HT-6) (Serotonin receptor 6)
GMEB1	bta02	Glucocorticoid modulatory element binding protein 1 (GMEB-1) (DNA binding protein p96PIF)
EGF	bta06	Epidermal growth factor (Urogastrone).
ERA	bta09	Estrogen receptor (ER) (Estradiol receptor) (ER-alpha)
PABPC1	bta14	Polyadenylate-binding protein 1 (Poly(A)-binding protein 1)
MFGE8	bta21	Lactadherin precursor (Milk fat globule-EGF factor 8) (MFG-E8) (HMFG) (Breast epithelial antigen BA46) (MFGM)
PRRP	bta26	Prolactin-releasing peptide receptor (PRRP receptor)

### Animals and DNA extraction

Exonic and intronic sequence was amplified from 15 genes in at least 1 individual from genomic DNA of the following breeds and species of cattle: Holstein (*B. taurus*), Tuli (*B. taurus*), Hereford (*B. taurus*), Banteng (*B. javanicus*), Gaur (*B. gaurus*), Yak (*B. grunniens*), Mithan (*B. frontalis*), Bison (*Bison bison*), Murrah buffalo (*Bubalus bubalis*), a nondescript breed of Indian River buffalo (*Bubalus bubalis*), East Asian River buffalo (*Bubalus bubalis*), a nondescript breed of Swamp buffalo (*Bubalus carabanesis*), Cape buffalo (*Syncerus caffar*) and Eland (*Taurotragus oryx*). For each animal genomic DNA was extracted from whole blood, semen or from tissue. Additional file 4 identifies the animals used in this study, their locality, date of extraction and a contact from which the material was sourced.

### Primer design and optimisation

*Bos taurus *coding sequence was extracted where available from Entrez at the NCBI . In the absence of *B. taurus *coding sequence *Homo sapiens *sequence was obtained and a reciprocal best hit algorithm (RBH) [43] was used to obtain the best orthologous cattle sequence from a large propriety in-house expressed sequence database (EST). An automated computer pipeline was used to identify and design primers for every exon from each candidate gene. Our program added forward and reverse M13 tails to all primer pairs in an effort to increase the throughput of the sequencing reactions [44]. Where possible primer pairs were designed to amplify 1000 base pair (bp) fragments. Amplicons typically contained 1 exon and a few hundred bp of intronic sequence.

All primer sets were optimised using a number of samples from the Bovidae. If optimisation failed to amplify any products a second primer pair was trialled. If no improvement was recorded, the amplicon was dropped from the analysis. A complete list of primers for all exons, a summary of PCR success in each species and Genbank accession numbers are available in additional file 4. Amplicons where all species amplified are available as a multiple alignment spanning the exon and flanking intronic sequences. This alignment was used extensively for phylogenetic reconstruction and is presented in additional file 2.

### PCR and sequencing conditions

For all primer pairs, PCR was performed in a 96 well plate format with 20 ul reactions. Each reaction contained 10–20 ng of DNA, 0.4 uM of forward and reverse primer, 20 uM dNTP Mix, 2.5 mM MgCl_2_, 10× reaction buffer and 0.5 U of Platinum Taq DNA polymerase. The conditions for amplification were as follows: 95°C for 10 min, 30 cycles of 95°C for 45 s and 60°C for 45 s followed by 95°C for 10 min with 30 cycles of 95°C for 45 s, 60°C for 45 s and 72°C for 30 s with a 90 min extension at 72°C.

Sequencing was completed using the Big Dye Terminator v 3.1 cycle sequencing kit on an ABI 3700 DNA Analyser (Applied Biosystems) according to the manufacturer's instructions for the sense and antisense strands. GeneScan version 3.7 (Applied Biosystems) was used to analyse the data collected from each capillary. Seqscape V. 2.5 (Applied Biosystems) was used to analyse chromatograms and resolve any potential heterozygous sequences. In total over 6,000 sequencing reactions were performed, analysed and exported as FASTA sequence files.

### Analysis

#### Consensus sequence generation

Sense and antisense reads were aligned using CLUSTALW [45] and used to create a consensus sequence for the analysis using an automated computer program. Consensus sequences for each amplicon were created from multiple alignments of sense and antisense reads for each species and breed. Choosing the most common residue at each point in the alignment creates the consensus, if one of two alleles does not occur at a frequency of 0.7 or higher, the ambiguous character (N) is selected, which masks polymorphisms within species and may result in a conservative estimate of the number of sites undergoing lineage sorting.

#### Ancient sequence generation

Alignments for each genomic fragment typically contained sequences representing the majority of the extant members of the tribe Bovini (Bovidae, Bovinae) and outgroup representatives from the tribes Tragelaphini (Bovidae, Bovinae) and Hominini (Hominidae, Ponginae). These alignments were used to create an ancient nucleotide sequence, which should represent the common ancestor of the Bovini tribe.

A computer program was developed to infer the ancestral state of each base between the two lineages leading to the two main subtribes of the Bovini, Bovina and Bubalina. The first subtribe Bovina includes all of the species from the genera Bos (cattle) and Bison (bisons) and the second subtribe Bubalina incorporates all of the species from the genera Bubalus (Asian buffalo) and Syncerus (African buffalo) (Table 1). The ancestral sequence for these two subtribes was inferred using Eland (Tragelaphini) and Human (Hominini) as outgroups. Wherever differences were detected between the two subtribes of Bovini the ancestral allele was inferred as the base that was shared between one of the subtribes and one of the outgroup species, the mutant or derived allele was assumed to be the unique allele with no other representatives in the phylogeny. Where both subtribes and the outgroup species had unique alleles the ancestral sequence was represented as an ambiguity character (N). Therefore, if j = a given position in the multiple alignment and Bv = Bovina subtribe, Bb = Bubalina subtribe, To = Eland, Hs = Human, An = Ancestral species and N = unknown base. Then if Bv(j) = Bb(j) An(j) = Bv(j), otherwise if Bv(j) ≠ Bb(j) and Bt(j) = Hs(j) or To(j) An(j) = Bt(j), or if Bb(j) = Hs(j) or To(j) An(j) = Bb(j), else if Hs(j) = To(j) An(j) = To(j); otherwise An(j) = N, where An(j) = the base at position j in the ancestral species.

For each amplicon ancestral sequences were aligned with consensus sequences from each member of the Bovini tribe. Large indels and ambiguous characters were removed manually from the alignment. Computer modules were written to undertake pairwise comparisons between all samples for the number of intronic substitutions per site (dI) and (non)synonymous substitutions per site (dN & dS). Exonic sequences and the corresponding open reading frame (ORF) were identified by cross-referencing alignments from known Bovine and Human protein coding genes [41]. All information per amplicon was summed up per gene and these values were used to estimate genetic distances between our samples.

#### Genetic distance, tree reconstruction and divergence times

Coding and noncoding regions were analysed separately for genetic distance calculations and phylogeny reconstruction. Base frequencies, substitution rates, transitions, transversions and genetic distances using an uncorrected p-distance were calculated for all sequences. Approximate divergence times (T_N_) were inferred using Nei's D from the intronic data for all pairwise comparisons with Hereford [25].

(6)

where t = the divergence time in years, μ = the mutation rate 2.2 × 10^-9 ^per year for mammals [46] and D is the genetic distance between species and breeds corrected for the level of Hereford polymorphism, which we assume is roughly similar to the levels of polymorphism in the common ancestor. So equation 5 should give estimates of the divergence times between species and breeds if dI is constant [46]. Due to T_N_'s reliance on an accurate mutation rate a second estimate for the time divergence was calculated (T_C_) was estimated using calibration points from the best estimates of divergence from fossil record for two nodes in the phylogeny. In the test we use Bison and Yak and the average divergence dates of 2.0 and 1.7 MY, respectively to calibrate the dates [16,47,48]. Phylogenetic trees were generated using the neighbour joining algorithm and Kimura's two-parameter distance using nonparametric bootstrapping with 5,000 replicates from the total (noncoding and coding) dataset in the MEGA4 package [25].

#### Identifying sites with abnormal inheritance in the Bovini

**Figure 4:**
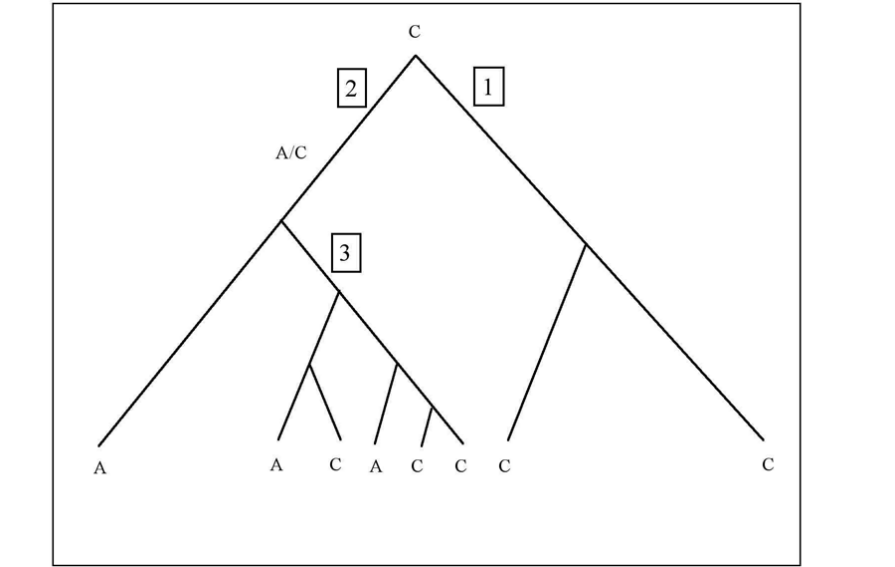
**A hypothetical monophyletic tree with three distinct clades (1), (2) and (3), where the A allele appears to have arisen in the extant species more than once**. This abnormal inheritance is due to either a homoplastic double mutation, a hybridisation event by two members of clade 2 with a member of clade 1 or lineage sorting has occurred on a homologous A/C polymorphism that arose in the most recent common ancestor (MRCA) of clades 1 and 2.

A computer program was generated to identify sites within introns and exons that differed from the proposed molecular phylogeny. The program identified aberrant sites that were either the result of a double mutation, recent gene flow between species or ancient polymorphisms. Hence, a site is aberrant under a given phylogenetic tree if the same mutation occurred twice or more on at least two different branches of a given phylogenetic tree (Figure 4). Derived alleles were inferred from the ancestral sequence and phylogenies were inferred from our and others' findings. Hence, if at position j in the alignment Mithan and Yak share an allele with Domestic cattle and Gaur, Banteng and Bison share an allele with the buffalo species, this site would be classified as an aberrant site. These aberrant sites are of interest, either because; they were polymorphic in an ancestral species and arisen due to lineage sorting, or they have arisen due to recent introgression, or are true homoplastic double mutations. The close relationship and limited divergence time of the Bovini subtribe make homoplasy (double mutations) highly unlikely. Thus, if we assume aberrant sites are not due to double mutations we should be able to test the accuracy of various phylogenetic reconstructions. By analysing the number of segregating sites within the dataset we were able to manually infer phylogenetic relationships and interrogate segregating sites for abnormal inheritance. Hence, the phylogenetic tree that minimised the number of abnormalities should add extra weight to the phylogenetic reconstruction inferred by our and others' studies.

#### Associations in the Bovini driven by lineage sorting or introgression

To identify whether lineage sorting or more recent introgression with domesticated cattle was responsible for any anomalies in genetic distance, an analysis was conducted examining genetic diversity between Domestic cattle and Mithan, Gaur, Bison and Yak at aberrant and all variable sites. Haplotype diversity was also examined between these species, with haplotypes resolved over all variable sites for each amplicon. Haplotypes generated from all variable sites were compared with *B. taurus *for 100% identity. Because Bovini subfamily is monophyletic all chromosomes originate from a common ancestor and therefore a high proportion of sites between all members of the Bovini are identical. Due to the geographic and sexual isolation of this clade many polymorphic sites are species or lineage specific, therefore identifying haplotypes that are 100% similar with *B. taurus *from variable single nucleotide sites are rare. To overcome this problem we examined haplotypes generated from homologous sites. Haplotypes at these sites should be more informative because they arose in the common ancestor, are present in all representatives of the clade and may be inherited abnormally and as a result may be responsible for confusing phylogenetic relationships. Also, due to the recent divergence of the Bovini tribe species-specific or double mutations at these exact sites are unlikely and haplotype structure should help identify recent introgression. Thus, if Bison or Yak have recently hybridised with Domestic cattle we would expect haplotypes generated from aberrant sites to be identical with those in *B. taurus *extended over amplicons in the same gene or possibly between genes on the same chromosome, while lineage sorting should show a more random mode of inheritance at these sites and consequently show low haplotype identity with *B. taurus *over the same region. However, care must be taken if haplotypes are generated over amplicons from only a few aberrant sites, as species may be identical by chance.

#### Ancient polymorphism and the neutral rate of evolution

All sites undergoing lineage sorting were differentiated as "old", which were defined as abnormally segregating sites in the Bovina subtribe, and "ancient", which were defined as any abnormal site that was still segregating in the Bubalina and Bovina subtribes. Classifying polymorphisms as either ancient or old should give insights into how long polymorphisms can persist in a species and its descendants. Aberrant sites that fell in the coding regions of the genes were identified, classified and the evolutionary ratio dN/dS was calculated [49].

## Competing interests

The authors declare that they have no competing interests.

## Authors' contributions

SM automated scripts for primer design and analysis, collected and sequenced all species and prepared the manuscript for submission. JM and MG coordinated the study and provided statistical and writing support. All authors read and approved the final manuscript.

## Supplementary Material

Additional file 1 **Supplementary table one of primers and amplification success for each amplicon in the study**. Summary of genes and exons where primer pairs have been designed for analysis and the success of each sequencing reaction. Click here for file

Additional file 2 **Total alignment concatenated from all Exonic and Intronic amplicons spanning shortest complete read**. Multiple alignment of Exonic and Intronic nucleotide sequence from which the majority of the analysis is based. Click here for file

Additional file 3 **Supplementary table two Aberrant allele positions and relative age**. Table summarising the position of all aberrant sites from figure 1 and the relative age of each of these sites (old = segregating since the divergence of Bubalina and Bos, ~8 MY). Click here for file

Additional file 4 **Supplementary table three Study animals localities**. Summary of animals used in this study, their locality, date of extraction and a contact from which the material was sourced. Click here for file
